# Effects of curcumol on ferroptosis and tube forming ability of hepatic sinus endothelial cells

**DOI:** 10.1515/med-2025-1338

**Published:** 2026-01-13

**Authors:** Jiahui Wang, Na Huang, Tiejian Zhao, Lei Wang, Yang Zheng, Huaye Xiao

**Affiliations:** Department of Medicine, Faculty of Chinese Medicine Science Guangxi University of Chinese Medicine, Nanning, China

**Keywords:** curcumol, P53-TFR1-FTH1 signalling axis, ferroptosis, angiogenesis, hepatic fibrosis

## Abstract

**Objectives:**

To explore the effects of curcumol on ferroptosis and angiogenesis of hepatic sinusoidal endothelial cells, further elucidate the molecular mechanism of curcumol against liver fibrosis, and provide new ideas for the prevention and treatment of chronic liver disease.

**Methods:**

We used VEGF to construct pathological model group, and divided hepatic sinusoidal endothelial cells into blank group, model group, high, middle and low curcumol group. Ferroptosis and angiogenesis were detected by various cell molecular biology experiments.

**Results:**

Curcumol significantly inhibited the proliferation and migration of hepatic sinusoidal endothelial cells, significantly increased the expression of P53 and TFR1 protein, significantly decreased the expression of FTH1 protein, significantly promoted the occurrence of iron death, and significantly inhibited angiogenesis. When we knocked out p53, the effect of curcumol contributing to the onset of ferroptosis was rescued, while curcumol’s role in inhibiting angiogenesis was saved, which was the same effect as when we used Ferrostatin-1.

**Conclusions:**

Curcumol targets the P53-TFR1-FTH1 signalling axis and induces massive deposition of iron ions in hepatic sinusoidal endothelial cells, leading to the onset of ferroptosis inhibiting hepatic angiogenesis, which may be one of the molecular mechanisms of its anti-hepatic fibrosis.

## Introduction

Long-term stimulation of the liver by a variety of pathogenic factors can produce persistent inflammation and spontaneous over-repair of tissues, prompting hepatic stellate cells to differentiate into myofibroblasts and secrete large quantities of extracellular matrix to accumulate in the liver, leading to the development of hepatic fibrosis [1]. Liver fibrosis is a necessary pathway for chronic liver disease to progress to refractory diseases such as cirrhosis or end-stage liver disease such as hepatocellular carcinoma [2]. There is no effective anti-hepatic fibrosis treatment in modern medicine, and the complexity of the pathogenesis of liver fibrosis makes it difficult for a single-target drug to be effective, and there is a lack of definitive therapeutic drugs for the treatment of liver fibrosis. Hepatic sinusoidal endothelial cells (HSEC), as the first group of cells to contact the hepatic blood flow, play an important role in maintaining the normal physiological function of the liver and preventing the development of liver diseases [3]. Normal hepatic sinusoidal endothelial cells have a unique fenestration structure on their surface, which facilitates the efficient exchange of substances between hepatocytes and hepatic blood sinusoids [4]. In the pathological state, the hepatic sinusoids were subjected to endotoxin, ethanol and oxygen free radicals, which resulted in a reduction in the diameter and number of HSEC window holes, accompanied by the formation of subendothelial basement membranes, leading to pathological neovascularisation characterised by capillarisation of the hepatic sinusoids [5], 6]. Pathological neovascularisation of the liver accompanies hepatic fibrosis, which is not only a pathological feature of hepatic fibrosis, but also one of the most important pathological reasons why hepatic fibrosis is difficult to be reversed, which occurs prior to hepatic fibrosis and continues throughout the development of hepatic fibrosis [7]. It has been shown that inhibition of pathological hepatic neovascularisation is effective in preventing the development of hepatic fibrosis [8]. Therefore, maintaining the normal phenotype of HSEC and inhibiting its pathological angiogenesis are important strategies to combat liver fibrosis. Therefore, inhibiting its pathological angiogenesis is an important strategy to combat liver fibrosis.

Ferroptosis is a new mode of programmed cell death first proposed by Dixon et al. in 2012, characterised by overloaded intracellular iron levels and oxidative damage [9]. Pathological iron accumulation can generate large amounts of reactive oxygen species (ROS) through the Fenton reaction, which reacts with polyunsaturated fatty acids on the cell membrane in an oxidative stress reaction, promoting the accumulation of toxic lipid peroxides and inducing oxidative cellular damage, leading to cellular ferroptosis [10]. Studies have shown that ROS is closely related to angiogenesis [11]. However, the relationship between ferroptosis of hepatic sinusoidal endothelial cells and angiogenesis has not been revealed. Our research group found that curcumol can regulate ferroptosis through multiple pathways and targets by means of systematic pharmacology [12]. We believe that the mechanism of action of curcumol against hepatic fibrosis has not been fully revealed. In the present study, we investigated whether curcumol affects pathological angiogenesis by mediating ferroptosis in hepatic sinusoidal endothelial cells.

## Materials and methods

### Reagents and antibodies

Curcumol was obtained from Aladdin Chemical Co., Ltd (Shanghai, China); Lipofectamine 2000 transfection reagent (Life Technologies, Grand Island, NY, USA); The siRNA-targeted for P53 and a negative siRNA were designed by Gene Pharma (Shanghai, China); Ferrostatin-1 purchased from sigmma-Aldrich (Sigma, USA); The following primary antibodies against P53, TFR1 and FTH1 were purchased from Abcam (Cambridge, UK); Antibodies SLC7A11, GPX4, CD31, ACSL4 and vWF were purchased from Cell Signaling Technology (Denver, MA, USA); VEGF cytokines purchased from sigmma-Aldrich (Sigma, USA); Phosphate Buffered Saline was obtained from Solarbio (Beijing, China).

### Cell culture and treatment

Mouse hepatic sinusoidal endothelial cells were purchased from procell company (Wuhan, China). Hepatic sinusoidal endothelial cells were mixed into complete medium containing 10 % fetal bovine serum (Sigma, St. Louis, MO, USA), inoculated into culture flasks, and placed in an incubator at 37 °C, 5 % CO_2_, and saturated humidity, and when the cells reached 80 % confluence, the cells were cultured in 12-well plates at a density of 2 × 10^5^ cells per well. The cells were divided into the following five groups: blank control group, model group, curcumol 12.5 mg/L group, curcumol 25 mg/L group, curcumol 50 mg/L group. The blank control group was cultured in Dulbecco’s Modified Eagle Medium (DMEM) supplemented with 0.1 % dimethyl sulfoxide (DMSO) (Invitrogen, CA, USA); the model group was incubated with 40 ng/mL VEGF for 48 h to activate the pathological model; and the curcumol groups were treated with 40 ng/mL VEGF for 48 h, followed by incubation with different final concentrations of curcumol for 48 h [13].

### Cell viability assay

The CCK8 kit (ab228554, Abcam, UK) was used to detect cell viability. Hepatic sinusoidal endothelial cells were inoculated into 96-well plates with 100 μL of approximately 5 × 10^3^ cells per well. After 24 h of incubation, the cells were removed from the plates, 100 μL of CCK8 solution was added to each well, and the incubation was continued for 4 h. The absorbance (A) value at 450 nm for each well was measured by enzyme marker (Thermo Fisher Scientific, USA).

### Transwell chamber invasion assay *in vitro*

In 24-well plates containing serum-free media (pore diameter 8 μm; American Corning), HSEC cells were injected in equal numbers. Complete media (500 μL) was introduced to the bottom chamber in the top chamber, and the cells were fixed with paraformaldehyde (Macklin, PA, USA) and stained with crystal violet after 24 h. Non-migrated cells in the top chamber were removed. Multiple locations in each well were randomly picked under the microscope (Olympus, Japan) for quantitative investigation.

### Western blot analysis

Cellular proteins were extracted, and their concentration was detected by bicinchoninic acid (BCA) (Thermo Scientific, CA, USA) with a protein marker (EpiZyme, Shanghai, China). Proteins were separated by sodium dodecyl sulfate polyacrylamide gel electrophoresis (SDS-PAGE), and electrotransferred to polyvinylidene fluoride (PVDF) membranes. Next, the membranes were incubated in 5 % skimmed milk powder at room temperature for 1 h. Primary antibodies were then added, including SLC7A11 (1:1,000), GPX4 (1:1,000), ACSL4 (1:1,000), CD31 (1:1,000), vWF (1:2000), P53 (1:1,000), TFR1 (1:1,000), FTH1 (1:1,000) and β-actin (1:1,000), and incubated overnight at 4 °C. The membranes were washed four times in tris-buffered saline with Tween (TBST), and incubated with a horseradish peroxidase secondary antibody (1:10,000) for another hour at room temperature. The grayscale values of the target bands were analyzed using ImageJ software, and the protein expression levels were normalized by the grayscale values of the internal reference (β-actin).

### Reverse transcription-quantitative polymerase chain reaction (RT-qPCR)

Total RNA was extracted with total RNA extraction reagent (Sigma, St. Louis, MO, USA), and primers were synthesized by Wuhan Bafeier Biotechnology Company with the help of Primer 5.0 software. According to the reverse transcription kit (Promega, USA), cDNA was synthesized. The reaction system was as follows: 95 °C for 1 min; 95 °C for 10 s, 60 °C for 30 s, 40 cycles, with GAPDH as the internal control, and the PCR products were taken for gel imaging analysis. The relative mRNA expression level of each sample=2^−△△Ct^. The primers are shown in Table 1.

**Table 1:** Primer sequence list.

Gene	Forward	Reverse
*SLC7A11*	5′-GGT​CCA​TTA​CCA​GCT​TTT​GTA​CG-3′	5′-AAT​GTA​GCG​TCC​AAA​TGC​CAG-3′
*ACSL4*	5′-CCC​ACT​TCA​GAC​AAA​CCC​G-3′	5′-GTA​TCT​GCT​CCA​GGG​ATG​TCT​A-3′
*GPX4*	5′-ATA​CGC​TGA​GTG​TGG​TTG​C-3′	5′-CTT​CAT​CCA​CTT​CCA​CAG​CG-3′
*CD31*	5′-GAG​TCC​TGC​TGA​CCC​TTC​TG-3′	5′-TCA​GGT​TCT​TCC​CAT​TTT​GC-3′
*VWF*	5′-GAG​GCT​GAG​TTT​GAA​GTG​C-3′	5′-CTG​CTC​CAG​CTC​ATC​CAC-3′
*GAPDH*	5′-GGT​TGT​CTC​CTG​CGA​CTT​CA-3′	5′-TGG​TCC​AGG​GTT​TCT​TAC​TCC-3′

### Immunofluorescence assay

Cells were fixed with 4 % paraformaldehyde (Sinopharm Group, China) at 37 °C for 60 min and then permeabilized by 0.1 % Triton X-100 (Beyotime Biotech Inc, China) for 10 min. Cells were blocked with 5 % bovine calf serum (Grand Island, NY, USA) for 30 min at room temperature and then incubated with indicated primary antibodies at 4 °C overnight. Fluorescence was developed by incubating with Cy3 or FITC (Beyotime Biotech Inc, China) labeled goat anti-rabbit IgG (Sigma, China) for 1 h at 37 °C. The nucleus was counterstained with DAPI (Beyotime Biotech Inc, China). Fluorescence image were captured under a fluorescence microscope (Japan Nikon Corporation).

### Prussian blue stain

Hepatic sinusoidal endothelial cells were removed from the culture medium after relevant treatment and rinsed twice with pre-warmed PBS. Fixed with 4 % paraformaldehyde for 10–20 min, incubated with Prussian blue (St Louis, MO, USA) staining solution in 37 °C for 30 min. Washing cells with deionized water for 2 times, re-dyeing with Prussian blue staining solution for 1 min, then re-washing it for 2–3 times. Finally, the blue particles were observed under the fluorescence microplate reader microscope (Japan Olympus Corporation).

### ROS measurements

The amount of ROS in six-well cell plates was assessed using oxidation-sensitive fluorescent probe DCFH-DA (Sigma, USA) and analyzed by flow cytometry (Beckman, USA).

### Detection of mitochondrial membrane potential

Cells were inoculated into 6-well cell culture plates, and after the treatment of each group of cells, the cells were collected by centrifugation, and 1 mL of JC-1 (Beyotime, China) staining working solution was added to each group of cells, which were incubated at 37 °C and protected from light for 30 min. After the incubation was completed, the cells were washed with PBS for 2 times and then resuspended, and detected by flow cytometry (Beckman, USA).

### Observation of cell ultrastructure by transmission electron microscope

Alterations in the mitochondrial structure of hepatic sinusoidal endothelial cells observed using transmission electron microscopy (Hitachi H-7000, Japan).

### Angiogenesis experiment

The 96-well plate was pre-cooled at 4 °C, 150 μL of Matrigel matrix gel (BD Biosciences, Bedford, MA) was added to each well and polymerised for 1 h in a 37 °C incubator and set aside, HSEC was inoculated into the wells covered with matrix gel. The angiogenesis was observed under a microscope after 8 h of incubation and analysed using Image J software (NIH, Bethesda, MD).

### Statistical analysis

IBM SPSS Statistics 25 was used for the statistical analysis (IBM, NY, USA). The means and standard deviations (SD) were used to express the data. One-way or two-way analysis of variance (ANOVA) was used to compare the groups, followed by Bonferroni post-hoc analysis. p<0.05 was considered statistically significant.

## Results

### Effects of curcumol on proliferation and migration of HSEC

**Figure 1:**
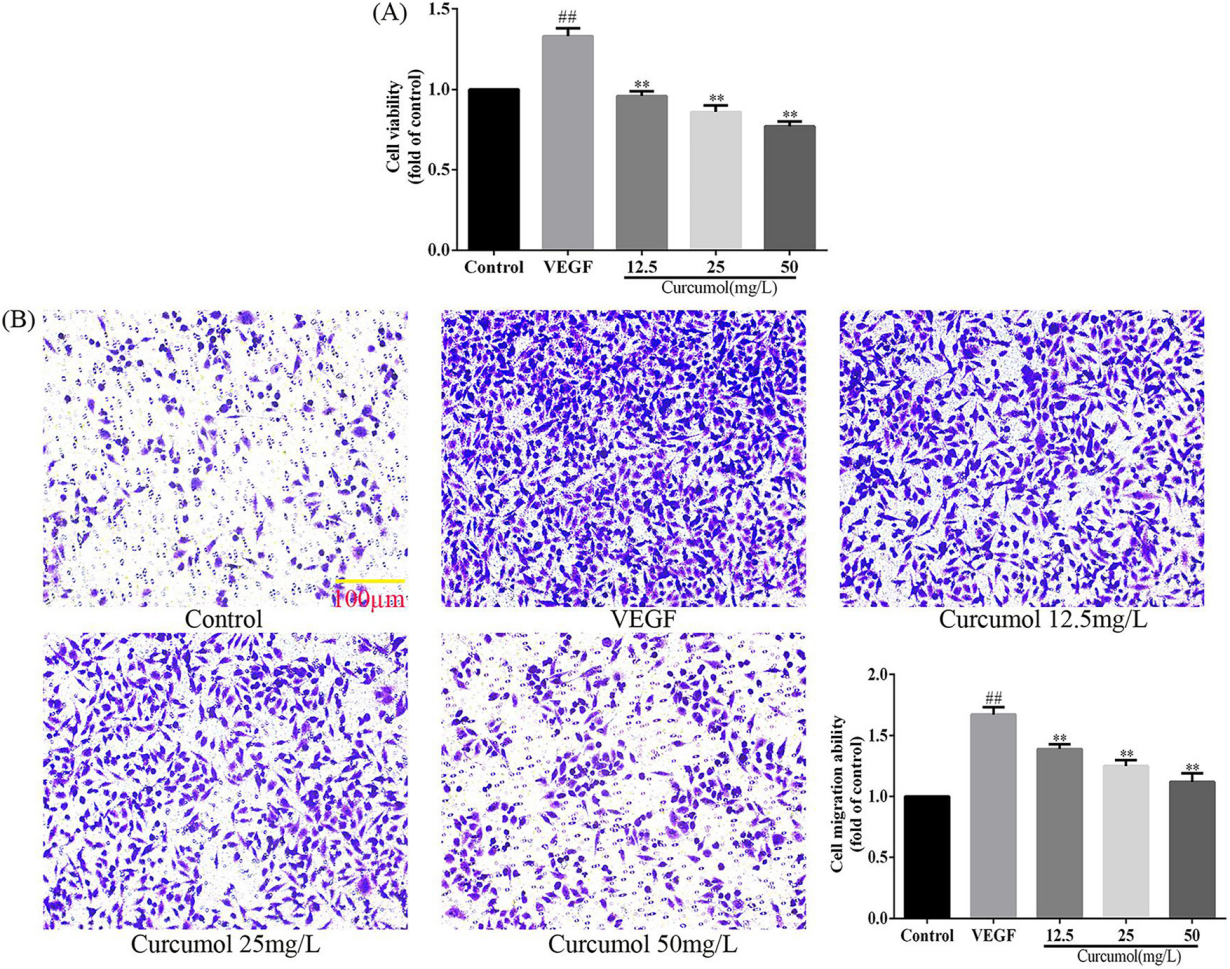
Effect of curcumol on HSEC phenotype. (A) Curcumol inhibits HSEC proliferation; (B) curcumol inhibits HSEC migration, scale bar=100 µm, for the statistics of each panel in this figure, data are expressed as mean ± SD, ^#^p<0.05 vs. control, ^##^p<0.01 vs. control, ^*^p<0.05 vs. VEGF, ^**^p<0.01 vs. VEGF.

To detect the effect of curcumol on the phenotype of HSEC, the proliferation and migration of HSEC were examined using CCK8 kit and Transwell assay. The CCK8 kit assay revealed a significant increase in HSEC proliferation rate after stimulation of HSEC with VEGF, and a significant decrease in HSEC proliferation rate when intervened with curcumol, as detailed in Figure 1A. The Transwell assay revealed a significant increase in HSEC migratory capacity after stimulation of HSEC with VEGF, and a significant decrease in HSEC migration capacity when intervened with curcumol, as detailed in Figure 1B. In conclusion, the inhibition of HSEC proliferation by curcumol may be the key mechanism by which it reduces the migration ability of HSEC.

### Effect of curcumol on signal axis of P53-TFR1-FTH1 in HSEC

**Figure 2:**
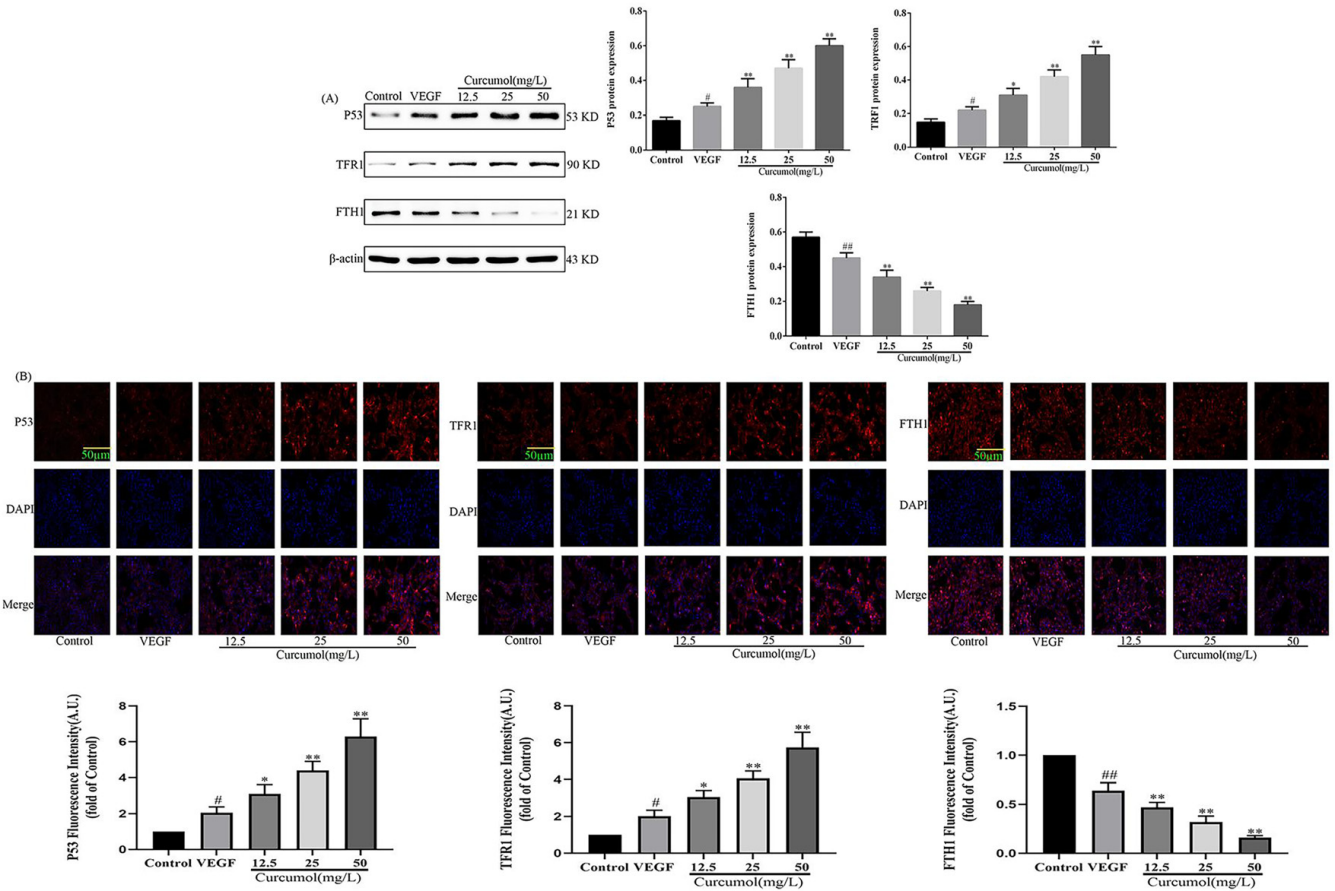
Effects of curcumol on the P53-TFR1-FTH1 signalling axis in HSEC. (A) WB detects the effect of curcumol on the P53-TFR1-FTH1 signaling axis, protein expression levels are normalized to β-actin; (B) immunofluorescence detects the effect of curcumol on the P53-TFR1-FTH1 signaling axis, scale bar=50 µm, for the statistics of each panel in this figure, data are expressed as mean ± SD, ^#^p<0.05 vs. control, ^##^p<0.01 vs. control, ^*^p<0.05 vs. VEGF, ^**^p<0.01 vs. VEGF.

To detect the effect of curcumol on the P53-TFR1-FTH1 signalling axis, the expression of P53, TFR1 and FTH1 was examined using WB and immunofluorescence. WB experiments revealed a significant increase in P53 and TFR1 protein expression and a significant decrease in FTH1 protein expression after VEGF stimulation of HSEC, and a significant increase in P53 and TRF1 protein expression after intervention with curcumol compared to VEGF stimulation, and a significant decrease in FTH1 protein expression compared to VEGF stimulation, as shown in detail in Figure 2A. Immunofluorescence experiments revealed that after VEGF stimulation of HSEC P53 and TFR1 protein expression increased while FTH1 protein expression decreased, and P53 and TRF1 protein expression increased after intervention with curcumol compared to VEGF stimulation, while FTH1 protein expression decreased compared to VEGF stimulation, as detailed in Figure 2B. Taken together, curcumol may induce lysosomal degradation of FTH1 by targeting P53 and TFR1 proteins, leading to the massive entry of iron ions into the cell.

### Effect of curcumol on ferroptosis of HSEC

**Figure 3:**
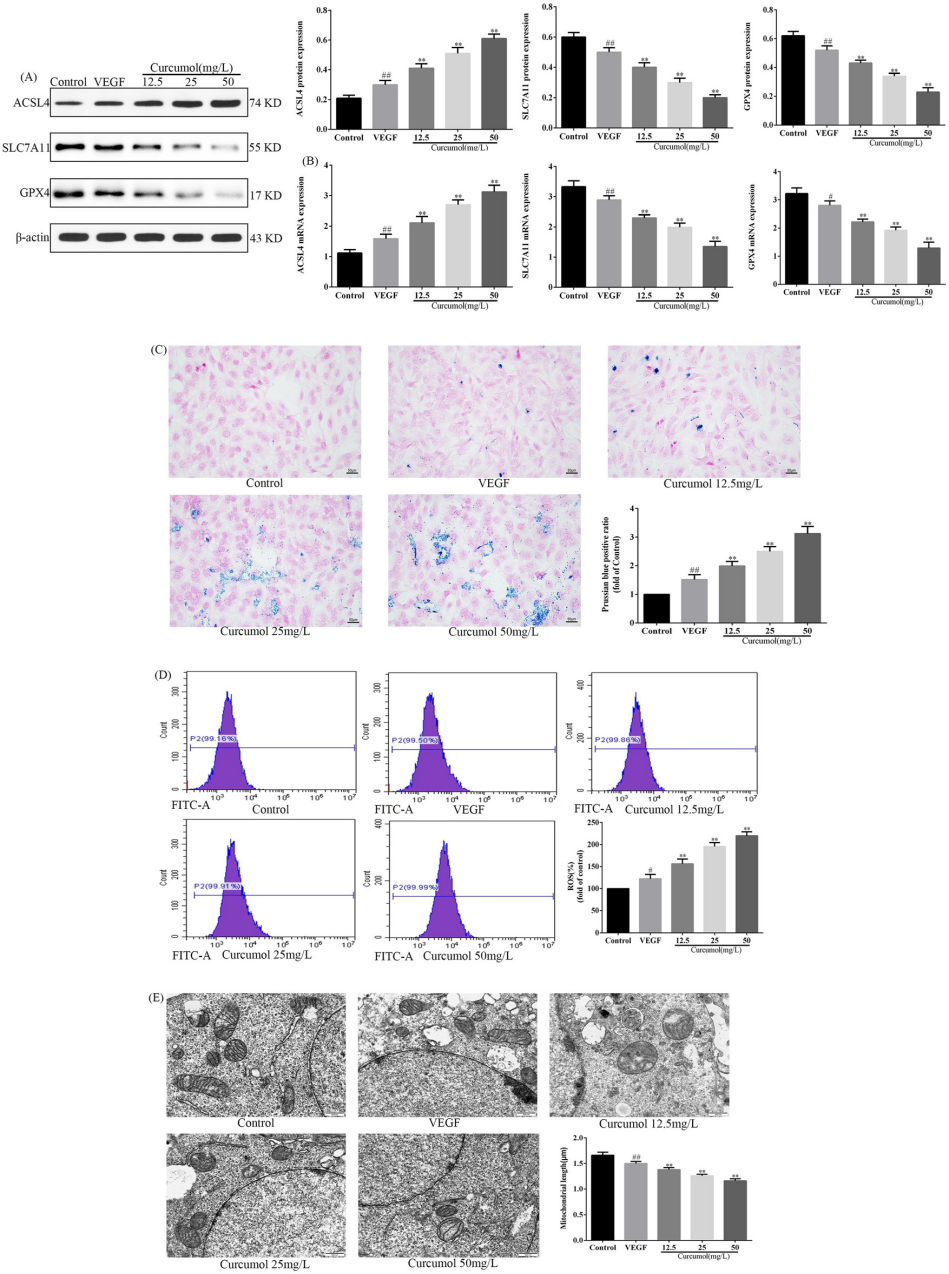
Curcumol induces ferroptosis in HSEC. (A) Effect of curcumol on the expression of ferroptosis-related proteins, protein expression levels are normalized to β-actin; (B) effects of curcumol on the expression of ferroptosis-related molecules mRNA, mRNA expression levels are normalized to *GAPDH*; (C) effect of curcumol on iron ion deposition, scale bar=50 µm; (D) effect of curcumol on ROS expression; (E) effects of curcumol on mitochondrial structure, scale bar=50 µm, for the statistics of each panel in this figure, data are expressed as mean ± SD, ^#^p<0.05 vs. control, ^##^p<0.01 vs. control, ^*^p<0.05 vs. VEGF, ^**^p<0.01 vs. VEGF.

To clarify the effect of curcumol on iron death in HSEC, WB and RT-PCR were used to detect the expression of ferroptosis-related molecules, as well as Prussian blue staining to observe the deposition of iron ions, fluorescent probes to detect ROS and transmission electron microscopy to examine mitochondrial structure. WB and RT-PCR experiments showed that curcumol significantly decreased the expression of SLC7A11 and GPX4 proteins, as well as *SLC7A11* and *GPX4* mRNA, whereas it significantly increased the expression of ACSL4 proteins and *ACSL4* mRNA, as detailed in Figure 3A and B. Prussian blue staining revealed that curcumol significantly increased the deposition of ferric ions in HSEC, as detailed in Figure 3C. Fluorescent probe detection of ROS expression revealed that curcumol significantly increased ROS expression, as detailed in Figure 3D. Transmission electron microscopy assay reveals that curcumol significantly reduces the length of mitochondria, as detailed in Figure 3E. In conclusion, curcumol induced ferroptosis in HSEC.

### Effect of curcumol on angiogenesis of HSEC

**Figure 4:**
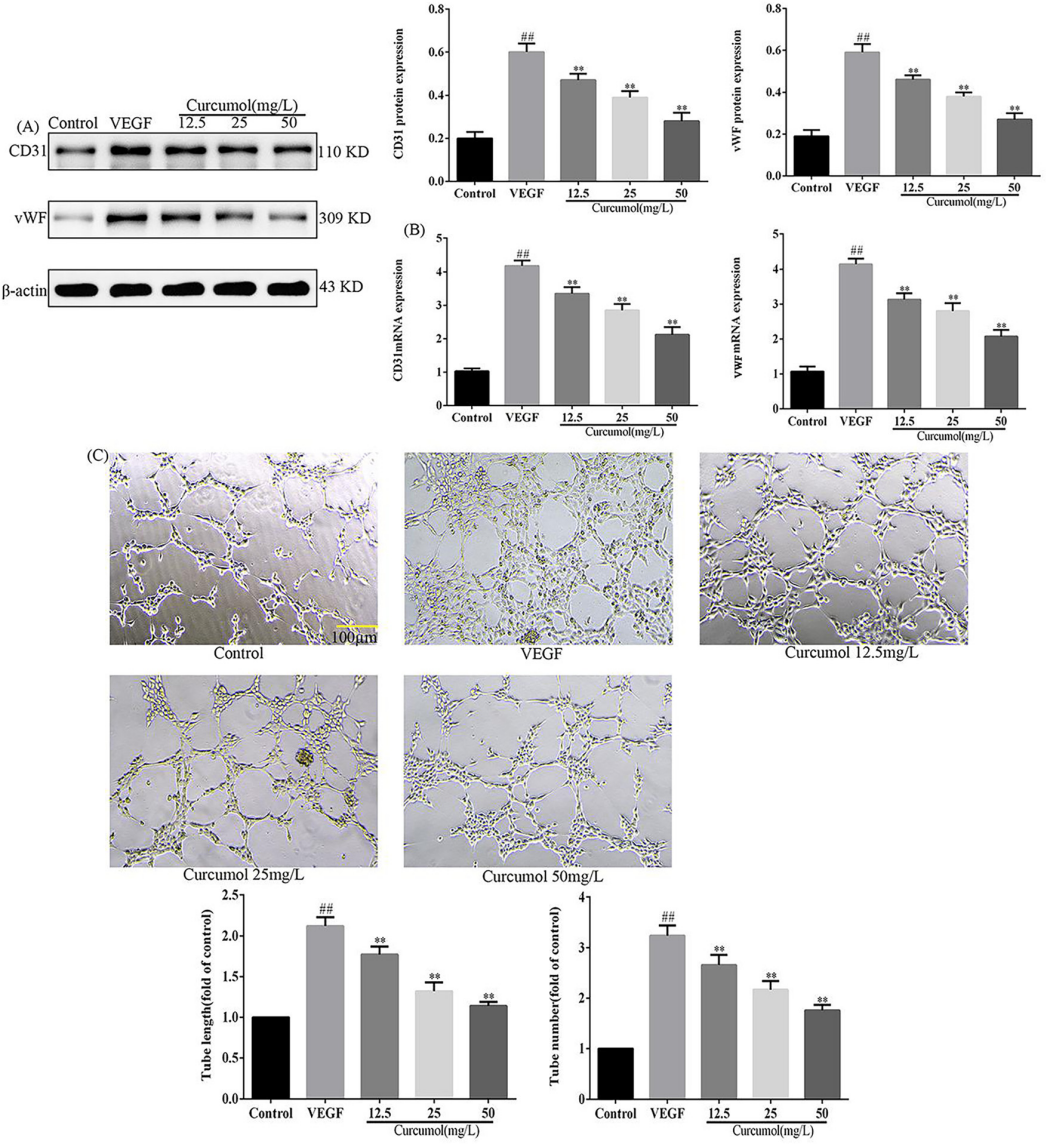
Effect of curcumol on angiogenesis. (A) Effects of curcumol on CD31 and vWF protein expression, protein expression levels are normalized to β-actin; (B) effects of curcumol on *CD31* and *VWF* mRNA expression, mRNA expression levels are normalized to *GAPDH*; (C) effect of curcumol on the number of angiogenesis, scale bar=100 µm, for the statistics of each panel in this figure, data are expressed as mean ± SD, ^#^p<0.05 vs. control, ^##^p<0.01 vs. control, ^*^p<0.05 vs. VEGF, ^**^p<0.01 vs. VEGF.

To clarify the effect of curcumol on HSEC-mediated neovascularisation, the expression of neovascularisation-related molecules was detected using WB and RT-PCR, and neovascularisation was observed using a neovascularisation assay. WB experiments showed that the expression of CD31 and vWF proteins was significantly increased after VEGF stimulation of HSEC, whereas the expression of CD31 and vWF proteins was significantly decreased after intervention with curcumol, and RT-PCR experiments showed that the expression of *CD31* and *VWF* mRNA was significantly increased after VEGF stimulation of HSEC, whereas the intervention with curcumol showed a expression of *CD31* and *VWF* mRNA was significantly reduced after intervention with curcumol, as detailed in Figure 4A and B. Angiogenesis experiments revealed a significant increase in the number of neovascularisations after VEGF stimulation of HSEC and a significant decrease in the number of neovascularisations when intervened with curcumol, as detailed in see Figure 4C. In summary, curcumol had an inhibitory effect on angiogenesis.

### Curcumol targets P53 to regulate ferroptosis mediated angiogenesis

**Figure 5:**
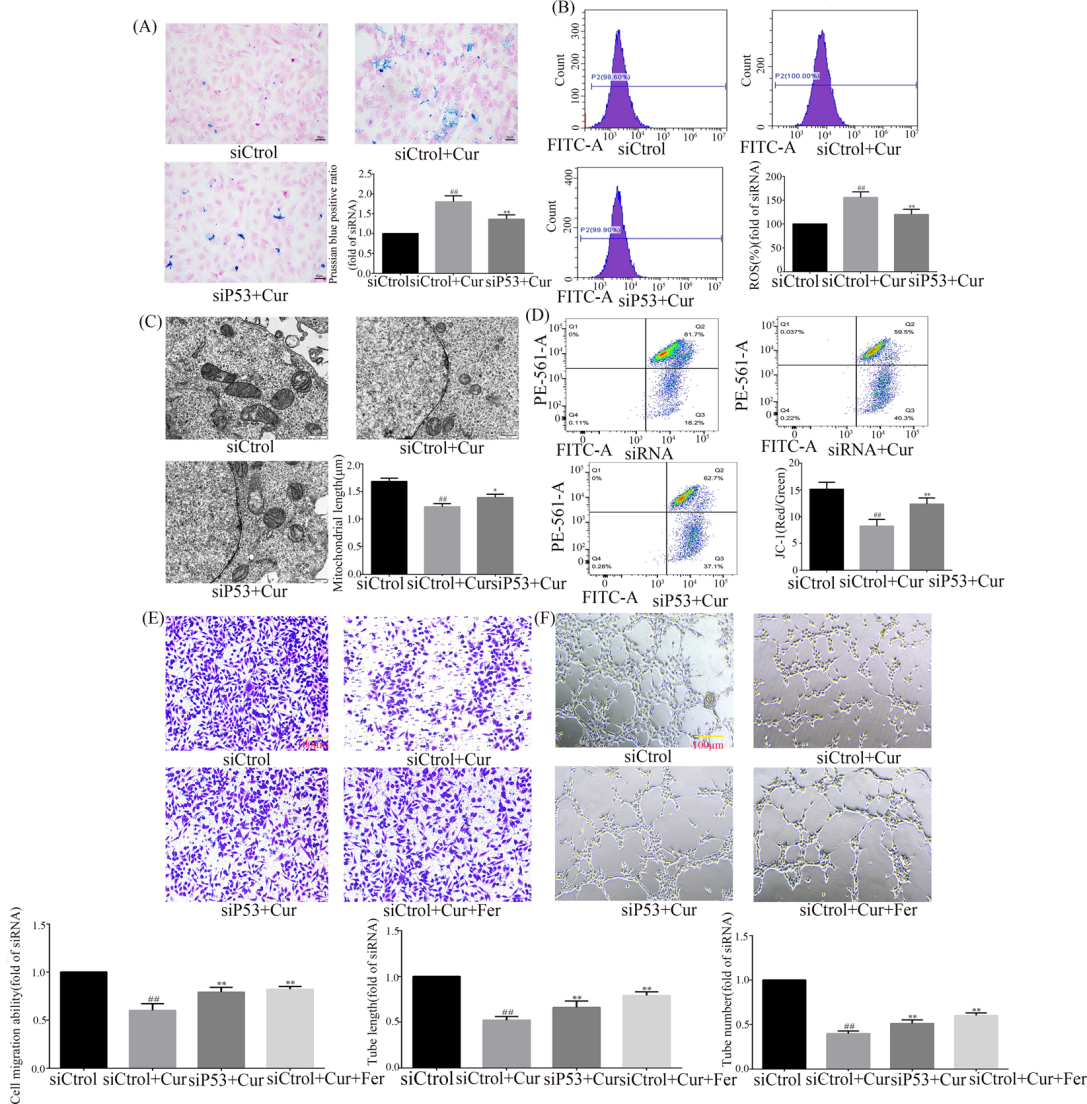
Curcumol targets P53 to regulate ferroptosis mediated angiogenesis. (A) Knockdown of P53 reverses curcumol-induced iron deposition; (B) knockdown of P53 reverses curcumol-induced ROS expression; (C) knockdown of P53 reverses the effect of curcumol on mitochondrial structure; (D) knockdown of P53 reverses the effect of curcumol on mitochondrial membrane potential; (E) effect of knockdown of P53 or Ferrostatin-1-reversal of curcumol on HSEC migration; (F) effect of knockdown of P53 or Ferrostatin-1-reversal of curcumol on angiogenesis, for the statistics of each panel in this figure, data are expressed as mean ± SD, ^#^p<0.05 vs. control, ^##^p<0.01 vs. control, ^*^p<0.05 vs. VEGF, ^**^p<0.01 vs. VEGF.

To investigate the targets of curcumol’s regulation of HSEC ferroptosis and to further clarify the mechanism of action of curcumol’s inhibition of angiogenesis. We performed functional recovery experiments using siP53 and Ferrostatin-1 after HSEC stimulation with VEGF. Ferrostatin-1 is a ferroptosis inhibitor that protects cells by inhibiting lipid peroxidation and prevents ferroptosis. Prussian blue staining revealed that curcumol-induced iron deposition was rescued by siP53, as detailed in Figure 5A. ROS assay experiments revealed that curcumol-increased ROS expression was rescued by siP53, as detailed in Figure 5B. Transmission electron microscopy revealed that curcumol-decreased mitochondrial length was rescued by siP53, as detailed in Figure 5C. Mitochondrial membrane potential assay revealed that curcumol-decreased mitochondrial membrane potential was rescued by siP53, as detailed in Figure 5D. The transwell assay revealed that the inhibition of HSEC migration by curcumol was rescued by siP53 or Ferrostatin-1, as detailed in Figure 5E. The angiogenesis assay revealed that the inhibition of angiogenesis by curcumol was rescued by siP53 or Ferrostatin-1, as detailed in Figure 5F. When Ferrostatin-1 was used, the inhibition of HSEC migration as well as the inhibition of neovascularization by curcumol was reversed, suggesting that ferroptosis is a key component of curcumol in exerting these pharmacological effects. Taken together, P53 is a key target of curcumol-induced HSEC ferroptosis, further suggesting that curcumol-induced HSEC ferroptosis is a key mechanism for its inhibition of angiogenesis.

## Discussions

Due to the prevalence of viral hepatitis and non-alcoholic fatty liver disease (NAFLD), liver fibrosis has become a very common disease in Asia and other parts of the world, increasing the healthcare burden on society [14]. Hepatic fibrosis occurs when the endothelial cells of the hepatic sinusoids, which are the main responding cells, continue to lose their window apertures and form a large number of contiguous basement membranes, a process known as hepatic sinusoidal capillarisation [15]. At the same time, the HSEC phenotype was greatly altered, expressing the vascular endothelial markers cluster of differentiation 31 (CD31) and von Willebrand factor (vWF) [16]. Studies have shown that hepatic sinusoidal capillarisation is the main form of microvascular neovascularisation in the liver [17]. Angiogenesis plays an important role in the pathological process of liver fibrosis characterised by collagen deposition and persistent inflammatory infiltration [18]. The intimate relationship between hepatic fibrosis and neovascularisation dictates that anti-neovascularisation can be an important strategy to halt and delay the progression of hepatic fibrosis.

Ferroptosis is an iron-dependent redox imbalance that leads to cell membrane phospholipid peroxidation damage [19]. Ferroptosis was manifested morphologically as a reduction in mitochondrial volume, an increase in membrane density, a decrease or disappearance of mitochondria, rupture of the outer membrane of some mitochondria, a significant decrease in mitochondrial cristae and rupture of the cell membrane [20]. The main biochemical characteristics of ferroptosis are the accumulation of iron and lipid peroxidation, which is manifested by the Fenton reaction between excess free Fe^2+^ and hydrogen peroxide, a product of mitochondrial oxidative respiration, resulting in a large increase in ROS, inhibition of glutathione (GSH) synthesis and glutathione peroxidase 4 (GPX4) activity, leading to impaired antioxidant system, redox imbalance, toxic lipid peroxidation, and impairment of the antioxidant system. The inhibition of GSH synthesis and GPX4 activity led to the impairment of the antioxidant system, redox imbalance, and the massive deposition of toxic lipid peroxides [21], 22].

Genetically, ferroptosis is regulated by a variety of genetic factors, focusing on both lipid peroxidation and iron homeostasis [23]. The cystathionine/glutamate reverse transporter protein solute carrier family 7 member 11 (SLC7A11) promotes cystathionine uptake and glutathione biosynthesis, and its up-regulation increases intracellular cystathionine levels and accelerates glutathione biosynthesis to enhance GPX4 activity, thereby preventing oxidative stress and ferroptosis [24]. Acyl-Co A synthetase long chain family member 4 (ACSL4) plays an important role in the synthesis of polyunsaturated fatty acids, which can modulate cellular resistance to ferroptosis [25]. Studies have shown that inducing ferroptosis in human venous endothelial cells can lead to inhibition of angiogenesis [26]. Further suggesting that ferroptosis is a key upstream signal for angiogenesis [27].

P53 is a transcription factor involved in the regulation of genome integrity, cell cycle arrest, apoptosis, autophagy, altered metabolism, cellular plasticity, and the promotion of iron-dependent cell death [27], 28]. P53 can reduce cystine and glutamate exchange by inhibiting SLC7A11, leading to a decrease in cysteine, the cystine reduction product of cystine in the cell, and a decrease in downstream GSH synthesis, which in turn reduces GPX4 activity, leading to the accumulation of ROS and thus inducing ferroptosis [29]. Fe^2+^ from food or erythrocyte degradation is oxidised to Fe^3+^ by plasma ceruloplasmin (CP), which binds to transferrin (TF) on the cell membrane to form TF-Fe^3+^, which enters the cell in a complex with TFR1, and is then reduced to Fe^2+^ by prostate transmembrane epithelial antigen 3 [30]. Intracellular iron ions can form a ferritin complex composition with ferritin heavy chains (FTH1) [31]. Nuclear receptor coactivator 4 (NCOA4) binds to FTH1 and subsequently transports the ferritin complex to autophagic vesicles for degradation and release of iron [32]. It has been shown that up-regulation of P53 expression, increase of TFR1 expression, and down-regulation of FTH1 expression lead to massive deposition of intracellular iron ions and induce the development of ferroptosis [33]. Taken together, targeting the P53-TFR1-FTH1 signalling axis is a key strategy to regulate ferroptosis.

In recent years, the study of natural active ingredients has gradually become an important direction in the development of drugs for the treatment of diseases. Curcuma comes from a variety of plants in the genus Curcuma of the ginger family. According to Chinese medicine theory, Curcuma is warm in nature, pungent and bitter in flavour, and belongs to the liver and spleen meridians. Modern phytochemical studies show that the chemical composition of *Curcuma longa* mainly includes volatile oil, curcumin, polysaccharides, sterols, phenolic acids and alkaloids [34]. The main components of the volatile oil of Curcuma are monoterpenes and sesquiterpenes such as curcumol, curcumin, curcuminone and β-elemene. Sesquiterpenoids possess various pharmacological activities such as antioxidant, anti-inflammatory and cytotoxic activities due to their structural specificity [35]. Curcumol is a natural product that structurally belongs to the guaiacol sesquiterpene family and has a wide range of biological activities. The anti-inflammatory and anticancer effects of curcumol have been extensively studied, and other therapeutic effects are being revealed [36], 37]. We have also recently found that curcumol has significant anti-hepatic fibrosis effects *in vitro* and *in vivo* [38], 39]. The above observations guided us to investigate whether the regulation of hepatic angiogenesis is related to the action of curcumol.

**Figure 6:**
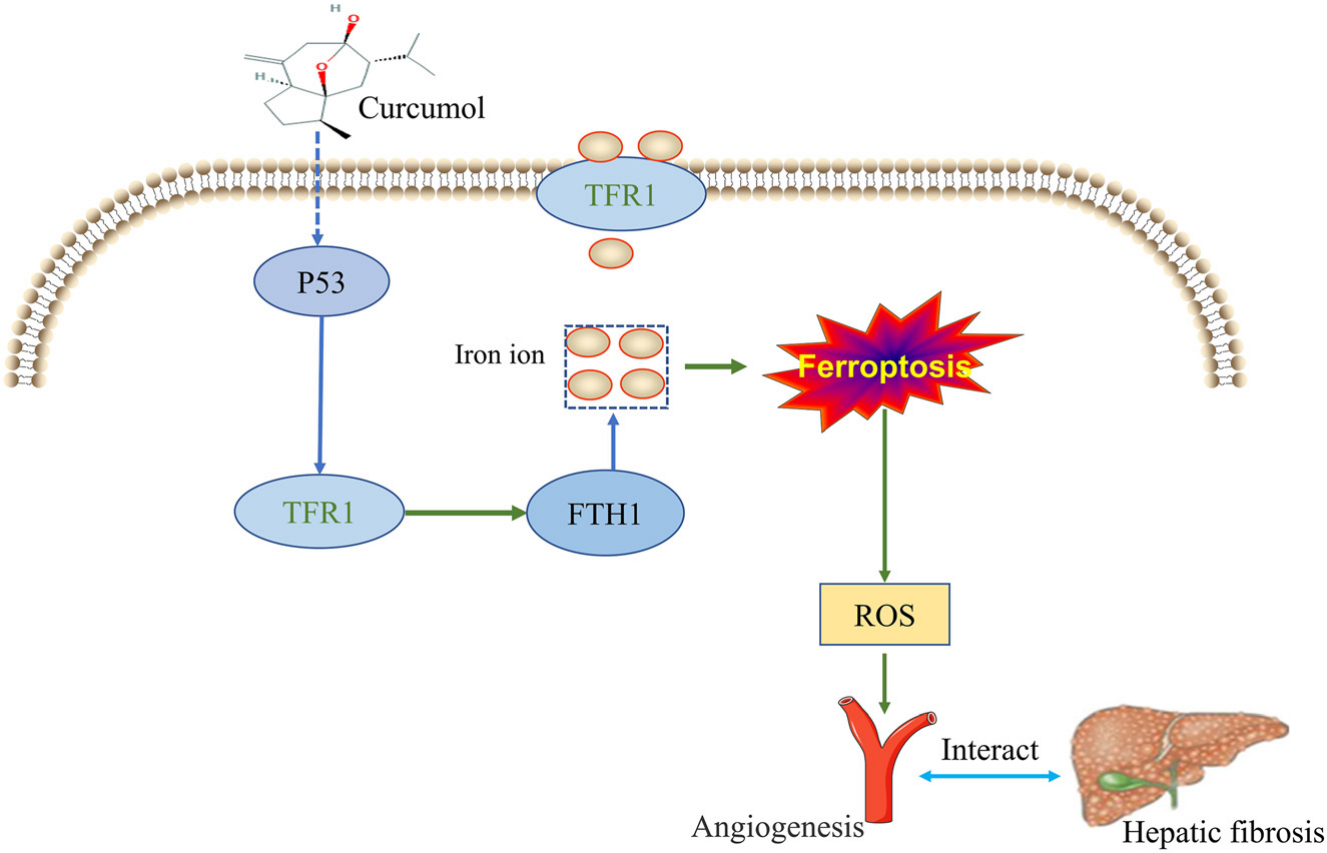
Curcumol targeting P53-TFR1-FTH1 signal axis induces ferroptosis in HSEC and inhibits angiogenesis.

In our study, we found that curcumol inhibited HSEC proliferation and migration. Meanwhile, curcumol can upregulate P53 and TFR1 expression, inhibit FTH1 expression, and induce HSEC to undergo ferroptosis, but the curcumol-induced ferroptosis of HSEC can be rescued by siP53, suggesting that P53 is a key target for curcumol to regulate HSEC. Further experiments revealed that curcumol had an inhibitory effect on angiogenesis, but this effect could be rescued by si P53 or ferroptosis inhibition, suggesting that the induction of ferroptosis is a key mechanism for curcumol to inhibit angiogenesis. However, it has now also been shown that inhibition of iron death inhibits angiogenesis, which is different from our reported results [40], 41]. The reason for this situation is related to the different drugs and cell types, but the most important thing is the degree of ferroptosis, because the degree of iron death occurs in different levels of ROS content, ROS in a certain dose range can promote angiogenesis, if ferroptosis continues to occur in large quantities of ROS deposition, will lead to apoptosis and inhibit angiogenesis [42]. Overall, the results suggest that curcumol inhibits angiogenesis by targeting P53-TFR1-FTH1 to mediate the deposition of large amounts of iron ions in HSEC and inducing the onset of ferroptosis in HSEC, as shown in Figure 6. These results compel us to investigate the complex mechanism between ferroptosis and angiogenesis. Of course there are some limitations in this study, such as no *in vivo* experiments were carried out, as well as no further clarification of the mechanism by which curcumol regulates P53 protein expression and how the increase in iron ions mediates the decrease in FTH1 protein expression. It was also not further clarified that P53 and iron death simultaneously play a role in angiogenesis.

## Conclusions

Our study suggests that causing ferroptosis is a key strategy for regulating angiogenesis. Regulation of iron metabolism mediated by the P53-TFR1-FTH1 signalling axis is an important mechanism for regulating angiogenesis. Curcumol induced large amounts of iron ions to be deposited in HSEC by targeting the P53-TFR1-FTH1 signaling axis, contributing to the inhibition of angiogenesis by ferroptosis in HSEC. Then, from the perspective of iron ion metabolism regulating angiogenesis, we elaborated the molecular mechanism of curcumol against hepatic fibrosis, which provides a new vision and a new target for the prevention and treatment of chronic liver diseases, and also further suggests that curcumol is a lead compound for anti-hepatic fibrosis.
